# A Multi-Factorial Genetic Model for Prognostic Assessment of High Risk Melanoma Patients Receiving Adjuvant Interferon

**DOI:** 10.1371/journal.pone.0040805

**Published:** 2012-07-24

**Authors:** Ena Wang, Yingdong Zhao, Alessandro Monaco, Lorenzo Uccellini, John M. Kirkwood, Maria Spyropoulou-Vlachou, Monica C. Panelli, Francesco M. Marincola, Helen Gogas

**Affiliations:** 1 Department of Transfusion Medicine, Clinical Center and Trans-NIH Center for Human Immunology, National Institutes of Health, Bethesda, Maryland, United States of America; 2 Division of Cancer Treatment and Diagnosis, National Cancer Institute, National Institutes of Health, Bethesda, Maryland, United States of America; 3 University of Pittsburgh Cancer Institute, Hillman Cancer Center, Pittsburgh, Pennsylvania, United States of America; 4 Department of Immunology, National Tissue Typing Center, General Hospital of Athens, Athens, Greece; 5 First Department of Medicine, University of Athens, Medical School, Athens, Greece; King’s College London, United Kingdom

## Abstract

**Purpose:**

IFNa was the first cytokine to demonstrate anti-tumor activity in advanced melanoma. Despite the ability of high-dose IFNa reducing relapse and mortality by up to 33%, large majority of patients experience side effects and toxicity which outweigh the benefits. The current study attempts to identify genetic markers likely to be associated with benefit from IFN-a2b treatment and predictive for survival.

**Experimental design:**

We tested the association of variants in FOXP3 microsatellites, CTLA4 SNPs and HLA genotype in 284 melanoma patients and their association with prognosis and survival of melanoma patients who received IFNa adjuvant therapy.

**Results:**

Univariate survival analysis suggested that patients bearing either the DRB1*15 or HLA-Cw7 allele suffered worse OS while patients bearing either HLA-Cw6 or HLA-B44 enjoyed better OS. DRB1*15 positive patients suffered also worse RFS and conversely HLA-Cw6 positive patients had better RFS. Multivariate analysis revealed that a five-marker genotyping signature was prognostic of OS independent of disease stage. In the multivariate Cox regression model, HLA-B38 (p = 0.021), HLA-C15 (p = 0.025), HLA-C3 (p = 0.014), DRB1*15 (p = 0.005) and CT60*G/G (0.081) were significantly associated with OS with risk ratio of 0.097 (95% CI, 0.013–0.709), 0.387 (95% CI, 0.169–0.889), 0.449 (95% CI, 0.237–0.851), 1.948 (95% CI, 1.221–3.109) and 1.484 (95% IC, 0.953–2.312) respectively.

**Conclusion:**

These results suggest that gene polymorphisms relevant to a biological occurrence are more likely to be informative when studied in concert to address potential redundant or conflicting functions that may limit each gene individual contribution. The five markers identified here exemplify this concept though prospective validation in independent cohorts is needed.

## Introduction

The incidence of melanoma in the US increased threefold in the past 3 decades [1] and an estimated 114,900 new cases were diagnosed in 2010; among them, 46,770 were noninvasive (*in situ*) while 68,130 were invasive resulting in 8,700 deaths [2]. The survival of patients, whose melanoma is detected early, is about 99 percent [3] and a 5 year survival of around 95% for stage I tumors [4]. However, the survival rate falls to 15 percent for those with advanced disease [5].

Interferon alpha (IFN-α) was the first cytokine to demonstrate anti-tumor activity in patients with advanced melanoma and is the only approved regimen for the adjuvant treatment for melanoma. Despite the ability of high dose IFN-α to reduce relapse and mortality by up to 33% [6], the large majority of patients experience side effects and toxicity which outweigh the benefits. Attempts to identify a subset of patients likely to benefit from adjuvant treatment with IFN-α2b have failed to discover clinical or demographic features of true predictive value. Correlative studies undertaken over the years identified a variety of immunologic parameters that are associated with therapeutic benefit but are observable only after therapy and, therefore, have no predictive value [7], [8]. Recently, mounting evidence indicates that immune cell infiltration in tumor correlates with prolonged survival suggesting a fine balance between tumor progression and immune recognition within the tumor microenvironment. This balance relies on multiple factors important for the maintenance of normal immune function. Among them, human leukocyte antigen (HLA), Cytotoxic T lymphocyte antigen 4 (CTLA4) and FOXP3, a member of the fork head/winged-helix family of transcriptional regulators are important first and secondary signal molecules influencing T lymphocyte activation and function. HLA genotype has been associated with disease susceptibility, immune responsiveness and prognosis [9], [10]. Single nucleotide polymorphisms (SNPs) in the CTLA4 and microsatellite polymorphism in FOXP3 gene region were also reported to be associated with several autoimmune diseases including, type-1 diabetes, systemic lupus erythematosus, autoimmune thyroid diseases and celiac disease [10]–[16]. Based on the observation that allelic polymorphism of individual genes is generally only weakly associated with disease predisposition in multi-genic disorders like cancer, a multi-factorial contribution by distinct genes affecting a specific function (i.e. T cell function) could be hypothesized; thus, polymorphisms of CTLA4, FOXP3 and HLA genes in melanoma patients may contribute to variability in patient survival and prognosis and their combination may have stronger predictive power than that of each genes assessed as a single entity. Thus, we tested the association of variants in FOXP3 microsatellites, CTLA4 SNPs and HLA genotype in 284 melanoma patients and their association with prognosis and survival of patients with melanoma who received adjuvant therapy with IFNa.

## Materials and Methods

### Study Subjects

The present translational research protocol was approved by the Bioethics Committee of the National and Kapodistrian University of Athens School of Medicine, Ethics Committee of Athens General Hospital “G. Gennimatas”, under the general title “Immunological studies of melanoma patients receiving adjuvant interferon” (A399/5-3-1999). Patients participating in this study were enrolled in Trial 13A/98, a prospective, multicenter, randomized phase III trial conducted at 13 institutions by the Hellenic Cooperative Oncology Group (HeCOG). In this trial, 364 patients with histologically documented AJCC stage IIB, IIC, or III primary cutaneous melanoma were enrolled between 1998 and 2004. For patients with clinically negative lymph nodes, stage was defined pathologically by sentinel lymph node (SLN) biopsy. Patients with positive SLN were required to undergo completion lymphadenectomy. All patients were randomized to receive treatment within 2 months of initial surgery or 1.5 months following therapeutic lymph node dissection. The regimen used was a modification of the E1684 regimen [17]. Group A patients received IFN-α2b (15 MIU/m^2^/day IV 5 days per week for 4 weeks) followed by observation. Group B patients received the same induction dose for 4 weeks followed by subcutaneous therapy (10 MIU/day TIW) for an additional 48 weeks. The primary endpoint for the core protocol was recurrence free survival (RFS) and overall survival (OS) and no difference in relapse free survival, distant metastasis free survival or overall survival was shown between the 2 groups [17].

The polymorphism study reported here was conducted retrospectively in four institutions that had participated in the core protocol. This study received separate IRB approval, and all patients had provided written informed consent for provision of biological material for such future research studies at initiation of treatment. Blood samples for CTLA-4, FOXP3 and HLA typing were drawn prior to treatment at the time of routine initial visit blood testing. The first 10 mL of blood were used for standard biochemistry and blood cell counts, and the second 3 mL were used for polymorphism testing. DNA was isolated using the BioRobot® EZ1 Genomic DNA Kit and (GenoVision, Oslo, Norway). The clinical outcome of patients was prospectively followed according to standard parameters; clinical staging consisted of medical history, physical exams, blood cell counts, blood biochemistry at 3-month intervals, and chest x-ray and liver ultrasound at 6-month intervals. Out of 364 patients, 284 were genotyped for all three genes assessed in the current study based on available DNA samples.

### Selection of Single Nucleotide Polymorphisms in the Human CTLA4 Gene and Microsatellite in FOXP3 Gene

Six CTLA4 SNPs, CT 60, AG 49, CT 318, JO 27, JO 30 and JO 31 were selected based on known association with autoimmune disorders [18]–[22]. CT 318 is located within the promoter region of the CTLA-4 gene, A/G49 is located at exon 1, while the rest of the SNPs are located at the 3′ untranslated region of CTLA-4 [22].

Microsatellite (TC)n of FOXP3 located within intron 5 from +476 to +595, up to +539 (IVS5) on Xp11.23 was selected based on previous publication and tested by using the previously described PCR primer pairs for primer sequences and nomenclature [14].

### Polymorphism Detection

HLA typing was performed using previously published DNA based techniques [23]–[25]. Initially HLA-A,-B,-Cw,-DRB1,and -DQB1 low resolution molecular typing was performed in all subjects, with amplification of genomic DNA by polymerase chain reaction (PCR) using locus-specific primers and reverse hybridization with sequence and allele-specific oligonucleotide probes (reverse PCR-SOP), using a commercially available kit (Lambda Array Beads Multi-Analyte System, LABType® RSS0, One Lambda, Inc). Subsequently, high resolution typing of HLA-DRB1 and -DQB1 loci was implemented by sequence specific oligonucleotide (SSO) (ELPHA HiRes, Biotest, Germany) and sequence specific primer (SSP) PCR (Olerup SSP™, Saltsjoebaden, Sweden) respectively. The allele assignment was made according to the HLA-visual software program.

FOXP3 (TC)_n_ microsatellite analysis was performed by capillary electrophoresis using DNA isolated from 259 patients. PCR amplification was conducted using (TC)n forward primer and reverse primer (Table 1). The reaction mixture were denatured at 95°C for 15 minutes and cycled 35 times at 94°C for 30 second, 54°C for 30 second and 72°C for 30 second followed by 72°C for 30 min. After digestion with EXOSAP at 37°C for 15 min and 80°C for 15 min to remove unincorporated primers and inactivate the enzyme, the PCR product mixed with internal size standard (Gene Scan-350) and formamide were analyzed by ABI Prism 3730 XL DNA analyzer. Data were analyzed using Genemapper software (Applied Biosystems) which automatically calls fragments size.

**Table 1:** PCR Primers for FOXP3 (TC)n amplification.

SNP name	Forward	Reverse
**CT 318**	5′-ACCCTTGTACTCCAGGAAATTCTC	5′-Biotin-GGTTTAGCTGTTACGTCGAAAAGA
**AG 49**	5′-TTTCAGCGGCACAAGGCTC	5′-Biotin-GAGTGCAGGGCCAGGTCC
**CT 60**	5′-GCAAGTCATTCTTGGAAGGTATC	5′-Biotin-TGCCAATTGATTTATAAAGGACTG
**JO 27**	5′-GAGCTGGTCAGCCGAGAT	5′-Biotin-TGACACCACCCCTCCATAAT
**JO 30**	5′-CAAAGCAAAACGCTGCCAATAA	5′-Biotin-TCCAGTGGCAATAGGAGCTTTC
**JO 31**	5′-TTGTCATGTTAGCCGTGCAGC	5′-Biotin-CCACCACCACACCCAGGTAA
**(TC)n**	5′-FAM-TCCACTGTTCCCAAAGTTCTAGC	5′GAGTGCTGGAGATAATGTTGGAAGT

 CTLA4 SNP-PCR was carried out with the primer pairs listed in Table 1. 50 ng of DNA were amplified in a 50 µL reaction containing 25 µL MasterMix (Illustra HotStart MasterMix, GE Healthcare, Buckinghamshire, UK) 1 µL (10 pmol) of each primer and nuclease free water. PCR were denatured at 5 minute at 95°C followed by 45 cycles of 95°C for15 seconds, 30 seconds at 56°C and 15 seconds at 72°C followed by final extension at 72°C for 5 minutes. The single strand PCR products were prepared by Streptavidin Sepharose™ High Performance beads capture (GE Healthcare, Uppsala, Sweden) after denaturation and washing (Biotage, Uppsala, Sweden) according to manual. All sequencing reactions were performed on the PyroMark™ ID pyrosequencer, using the PSQ 96 SNP Reagent Kit (Biotage AB), under sequence primer (Table 1) and analysis was done with PyroMark™ ID 1.0 software.

### Data Processing and Statistic Analysis

#### Variable coding

For HLA alleles, each patient was counted as an event and a given allele was coded as 1 if detected either in heterozygous or homozygous conditions or 0 if undetected. For each allele, an additional code classified as 1 for homozygosity and 0 for heterozygosity. For CTLA-4 and FOXP3 testing, each patient was counted as an unique event and a particular allele was coded as 1 if detected or 0 if undetected. Of all potential variant alleles only those presented at least with a frequency of 10%, i.e., a total of 71 variables were included in the analysis.

#### Feature selection and prediction models

OS and RFS were calculated from the date treatment was started to the date of last follow-up or the date when death from any cause or relapse first occurred. We first performed univariate analysis to identify markers (alleles) whose presence/absence correlated with survival by fitting Cox proportional hazards model [26] that computed the p value for each allele testing the hypothesis that survival was independent of the presence/absence of that allele. This backward elimination method was used for feature selection in the multivariate Cox proportional hazard model. A prognostic index for each patient was calculated as the weighted average (weighted as regression coefficient) of the variables selected for the multivariate Cox model. A high value of the prognostic index corresponded to a high hazard of death, and consequently a poor predicted survival. The patients were classified into high risk or low risk groups based on whether their prognostic index was above or below the median.

Leave-One-Out-Cross-Validation (LOOCV) was applied to evaluate the predictive accuracy of survival risk classifiers based on high-dimensional data [27]. A single event (patient) was omitted and the entire procedure described above was performed to create a prognostic index. This function was created from scratch on the training set with the one case removed, including the feature selection step in the multivariate Cox model. Having determined a prognostic index function for that training set, this was used to compute a prognostic index for the omitted event. That observed value was compared to the prediction of the prognostic index for the n-1 cases included in that training set. The omitted patient was placed into either a high risk group or a low risk group based on his/her prognostic index >  =  or < the median of the prognostic index in the training set. This analysis was repeated from scratch n times, leaving out a different patient each time.

Kaplan-Meier survival curves were plotted for the cases predicted to bear above or below average risk as for the above model. Since the risk group for each case was determined based on a predictor that did not use that case in its construction, the Kaplan-Meier curves were unbiased and the separation between the curves gave a fair representation of the predictive value of genotyping profiles on survival.

Next, we tested whether the association of genotyping with survival was statistically significant. A log-rank statistic (LR_d_) was computed for the cross-validated Kaplan-Meier curves. We performed a statistical significance test by randomly shuffling survival data among the cases and repeating the entire cross-validation process. For each random re-shuffling, we repeated the process, created new cross-validated Kaplan-Meier curves, and computed the log-rank statistic for the random shuffling, which served as a null-distribution of the log-rank statistic created in this way. We defined the tail area of this null distribution beyond the value LR_d_ obtained for the real data as the permutation significance level for testing the null hypothesis that there was no relation between the genotyping data and survival.

We then compared the combined survival risk model (i.e., genotyping + covariate) to the model based only on the covariate (i.e., stage) using as a test statistic the difference between the cross-validated log-rank statistic for the combined model minus the log-rank statistic for cross-validated Kaplan–Meier curves for the covariate model. The null distribution of the test statistic was generated based on permuting the genotyping vectors among cases. In these permutations, the correspondence between survival times, censoring indicators and the covariate were not disrupted. The null hypothesis tested was that the genotyping data were independent of survival and the covariate. This approach is described more fully in R Simon et al. [27].

We finally evaluated if the survival risk model built on OS data can be used to predict RFS. LOOCV was performed by building the survival risk model on the training set OS data but validating the model on the test set RFS data.

Considering the arbitrary cut-off percentiles specified for defining the risk groups (50^th^ percentile cut-off was used in our case), we created a time-dependent ROC curve from censored survival data [28], indicating how well the marker predicted the survival time for the subjects in the dataset by TP (True Positive), FP (False Positive), AUC (Area Under (ROC) Curve) at the time point of interest (e.g., 7 year survival).

## Results

### Individual Genotyping Markers Predict OS and RFS of Patients with Melanoma Receiving Adjuvant IFN-α Therapy

**Figure 1:**
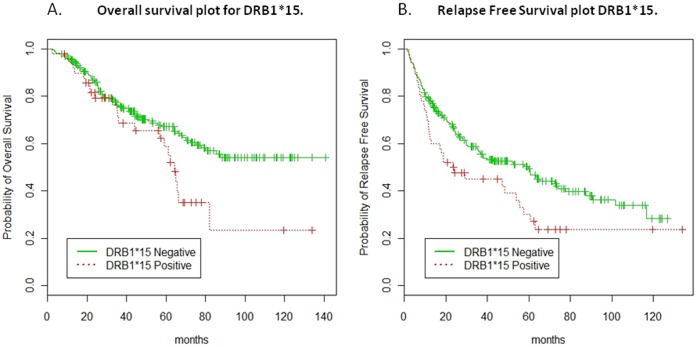
Kaplan-Meier curves for DRB1*15 for OS and RFS. The Leave-One-Out Cross Validated (LOOCV) Kaplan-Meier curves to compare DRB1*15 positive (red line) and negative patients (green line) for OS (log rank p value = 0.027) (Figure 1A) and RFS (log rank p value = 0.025) (Figure 1B), respectively.

Univariate survival analysis suggested that patients bearing either the DRB1*15 or HLA-Cw7 allele suffered worse OS while patients bearing either HLA-Cw6 or HLA-B44 enjoyed better OS. DRB1*15 positive patients suffered also worse RFS and conversely HLA-Cw6 positive patients had better RFS. The p values, Hazard Ratio (HR), and 95% Confidence Interval (CI) of HR for the significant markers (Wald test p value <0.05) are listed in Table 2 and Table 3, for OS and RFS, respectively. Kaplan-Meier curves for DRB1*15 are shown in Figure 1A and 1B for OS (log rank p value = 0.027) and RFS (log rank p value = 0.025), respectively. Comparison of FOXP3 microsatellites did not demonstrate significant associations (Univariate survival analysis for FoxP3 microsatellites, Table S1) and, therefore, the final data presented here using a model excluded FOXP3 from the analysis. We would however emphasize that this results should not exclude completely lack of association between FOXP3 genetic variants and outcome of IFN-α therapy as other SNPs not analyzed in this study could have provided different information. Such analysis is the subject of future studies.

**Table 2:** Univariate overall survival (OS) analysis.

Variables	P value	HR	95% CI of HR
DRB1*15	0.028	1.676	[1.056, 2.659]
Cw6	0.029	0.510	[0.279, 0.933]
Cw7	0.030	1.547	[1.044, 2.291]
B44	0.040	0.421	[0.184, 0.961]

**Table 3:** Univariate relapse free survival (RFS) analysis.

Variables	P value	HR	95% CI of HR
Cw6	0.021	0.593	[0.380, 0.924]
DRB1*15	0.026	1.543	[1.054, 2.260]

### A Five-marker Genotyping Signature has Prognostic Significance on OS of Patients with Melanoma Receiving Adjuvant Therapy with IFN-α

Multivariate analysis revealed that a five-marker genotyping signature was prognostic of OS. In the final multivariate Cox regression model, HLA-B38 (p = 0.021), HLA-C15 (p = 0.025), HLA-C3 (p = 0.014), DRB1*15 (p = 0.005) and CT60*G/G (0.081) (Table 3) were significantly associated with OS with risk ratio of 0.097 (95% CI, 0.013 to 0.709), 0.387 (95% CI, 0.169 to 0.889), 0.449 (95% CI, 0.237 to 0.851), 1.948 (95% CI, 1.221 to 3.109) and 1.484 (95% IC, 0.953 to 2.312) respectively (Table 4). A prognostic index for a patient with a specific genotyping profile could be calculated as the weighted average (weighted as regression coefficient) of the above five variables.

**Table 4:** Multivariate overall survival (OS) analysis.

Alleles	HR	95% CI of HR	P value
**HLA-B38**	0.097	[0.013, 0.709]	0.021
**HLA-C15**	0.387	[0.169, 0.889]	0.025
**HLA-C3**	0.449	[0.237, 0.851]	0.014
DRB1*15	1.948	[1.221, 3.109]	0.005
CT60*G/G	1.484	[0.953, 2.312]	0.081

**Figure 2:**
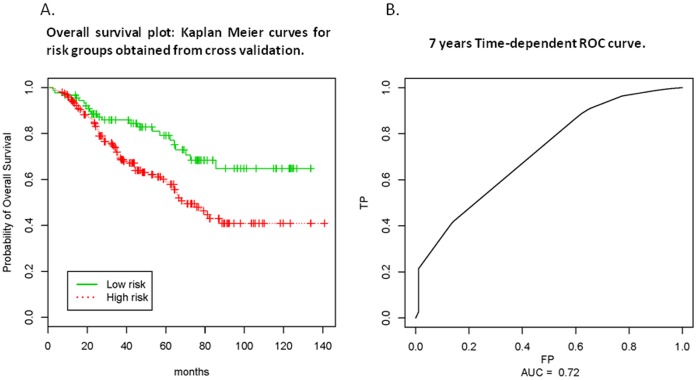
LOOCV Kaplan-Meier and Time-dependent ROC curves. A prognostic index for a patient with a specific genotyping profile were calculated as the weighted average (weighted as regression coefficient) of the five-marker genotypes. LOOCV Kaplan-Meier curve (Figure 2 A) identified 90 patients (22 deaths) in the low risk group (green line) and 194 (78 deaths) in the high risk group (red line). Time-dependent ROC curves showed that the AUCs at 5 and 7 year were 0.645 and 0.720, respectively (Figure 2B, 7 year survival ROC curve).

LOOCV Kaplan-Meier curve (Figure 2 A) identified 90 patients (22 deaths) in the low risk group and 194 (78 deaths) in the high risk group. The median OS of the low risk group has not yet been reached, while it was 68.2 months in the high risk group (log rank test p = 0.0026). The permutation p-value was 0.04 (500 permutations) indicating that the association of genotyping with OS was statistically significant. Time-dependent ROC curves showed that the AUCs at 5 and 7 year were 0.645 and 0.72, respectively (Figure 2B, 7 year survival ROC curve).

### A Five-marker Genotyping Signature is an Independent Predictor of OS Independent of Disease Stage

To assess whether the prediction of OS by the five marker signature was affected by stage of disease, multivariate Cox regression analysis was performed by adding stage as a factor. Hazard ratios (HR), 95% CI of HR, and p values are shown for the five markers and disease stage (Table 5).

**Table 5:** Multivariate overall survival (OS) analysis with stage correction.

Alleles	HR	95% CI of HR	P value
**HLA-B38**	0.089	[0.012, 0.648]	0.017
**HLA-C15**	0.443	[0.192, 1.020]	0.056
**HLA-C3**	0.446	[0.235, 0.847]	0.014
DRB1*15	1.729	[1.078, 2.773]	0.023
CT60*G/G	1.715	[1.093, 2.691]	0.019
**Stage**	2.062	[1.314, 3.234]	0.002

**Figure 3:**
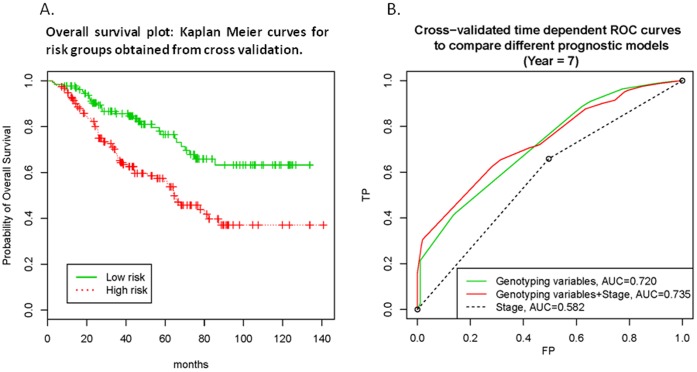
Multivariate Cox regression analysis with stage as a factor. LOOCV Kaplan-Meier curve (Figure 3A) showed that 131 patients (31 deaths) were in the low risk group (green line) and 153 patients (69 deaths) were in the high risk group (red line). The Leave-One-Out Cross Validated time dependent (7 year) ROC curves (Figure 3B) to compare different prognostic models: Stage only (dashed line); Stage and five-marker genotyping signature (red line); Five-marker genotyping signature only (green line).

LOOCV Kaplan-Meier curve (Figure 3A) showed that 131 patients (31 deaths) were in the low risk group and 153 patients (69 deaths) were in the high risk group. The median OS of the low risk group had not yet been reached, while it was 64.3 months in the high risk group (log rank test p = 0.00009). The permutation significance level was 0.05 (500 permutations), indicating that the association of genotyping data with OS data was statistically significant. Time-dependent ROC curve showed that the AUC for 7 year survival was 0.735. This, the five-marker genotyping signature was an independent predictor of OS after controlling for disease stage (Figure 3B) while stage of disease was an independent risk factor (Table 3).

### A Five-marker Genotyping Signature may be Used as a Prognostic Index for RFS in Patients with Melanoma Receiving Adjuvant IFN-α Treatment

We attempted to build the RFS model using an approach similar to the OS model. However, Kaplan-Meier plot obtained from LOOCV did not show a clear separation between the curves, indicating a poor performance of the model in predicting RFS risk.

**Figure 4:**
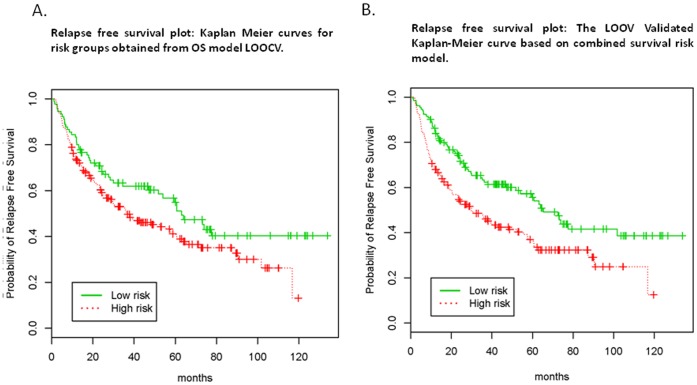
The LOOV Validated Kaplan-Meier curve based on combined survival risk model. The LOOCV Kaplan-Meier curves showed that the five-marker OS model (Figure 4A) and the combined model (five-marker + stage model) (Figure 4B) may be used to predict RFS survival data. In each round of LOOCV, the model was built in the training set using the OS information and tested on the left out sample using the RFS information.

We then evaluated the five-marker OS model on RFS survival data. LOOCV was performed as predictive of OS but then was tested on RFS survival data. The LOOCV Kaplan-Meier curve demonstrated that 90 patients (44 relapses) were in the low risk group (median RFS: 36.7 months) and 194 patients (111 relapses) were in the high risk group (median RFS: 63.0 months) (log rank test p = 0.048) (Figure 4A). The permutation significance level was 0.09 (1,000 permutations), indicating the association of genotyping data to RFS data was borderline statistically significant.

We finally evaluated if the combined survival risk model, i.e., genotyping + stage model built on OS data, can also be used to predict RFS. The Leave-One-Out Cross Validated Kaplan-Meier curve is shown in Figure 4B, with 131 patients (60 relapses) in the low risk group (median RFS 29.9 months) and 153 patients (95 relapses) in the high risk group (median RFS 65.3 months) (log rank test p = 0.001). The permutation significance level was 0.06 (500 permutations), indicating the association of genotyping data and stage to RFS data was borderline statistically significant.

## Discussion

In the past decade, great efforts have been dedicated to identify and validate prognostic markers in patients with advanced melanoma. Such markers could assist the selection by physicians of the most suitable treatments on a patient-specific basis. In the case of IFN-α therapy, although robust predictors of response to therapy have not yet been identified weak prognostic associations have previously been described [29]. Recently, increased infiltration of lymphocytes within tumors was recognized to bear positive predictive value and correlate with good prognosis in patients with various cancer types independent of treatment suggesting an important role of the adaptive immune response in controlling disease progression [30]–[33]. This may in turn bear predictive and prognostic significance in patients with melanoma treated with IFN-a as this cytokine is believed to exerts its effects at least in part through activation of T cells within the tumor microenvironment [34]. Therefore, polymorphism of genes involved in the modulation of T cell function could play important role in promoting or inhibiting cancer progression. It has been reported that IFN-γ (+874A–T) polymorphism is significantly associated with RFS and OS of patients with melanoma. Combined with two weakly or marginally associated polymorphisms of IL10 and ERCC1, patients could be stratified into distinct groups with different clinical outcomes [35]. Furthermore, polymorphism of CCR5, a chemokine receptor preferentially expressed by Th1 T cells and responsible for homing to the site of inflammation via interaction with its ligands has been reported to have clinical relevance; the Δ32 deletion of CCR5 was reported to be associated with decreased survival in patients with melanoma receiving immunotherapy [36]. A combination of three polymorphism of CDKN2A spanning the coding exon 1a (rs2811710), the first intron (rs2518720), and the *5′* regulatory region (rs2811708) was also reported significantly associated with decreased OS in melanoma [37].

In the current study, CTLA4, FOXP3 and HLA were selected because T lymphocytes play a major role both in tumor immunity and autoimmunity and these genes are well known to directly or indirectly affect T cell function. CD28, CTLA4 and inducible co-stimulator (ICOS) molecules are key secondary signal molecules in T lymphocyte activation. SNPs in the CD28/CTLA4/ICOS gene region were reported to be associated with several autoimmune diseases including, type-1 diabetes, systemic lupus erythematosus, autoimmune thyroid diseases and celiac disease [11], [20], [38]. High expression of FOXP3 in CD4+ and CD25+ T helper cells is indicative of immune suppressive function [39], [40]. However, recent publications indicate that increased FOXP3 expression in CD8+ T cell serves as a good prognosis marker in melanoma patients undergoing high does IL-2 combined with peptide vaccination therapy [41]. We observed that FOXP3 gene up regulation in tumor infiltrating lymphocytes expanded in vitro for adopted transfer therapy is associated with likelihood of response to therapy (unpublished observation). Correlation of HLA phenotypes with clinical outcome have been studied in infectious disease, transplantation rejection, disease susceptibility and prognosis of cancer [42].

Previously, we reported that RFS and OS did not differ significantly between patients with distinct CTLA4 polymorphisms when assessed alone [12]. Similarly, individual HLA class I and II alleles were not informative in predicting recurrence in patients receiving adjuvant IFN-α with the exception of HLA-Cw*06 which was associated with a better RFS and OS [43]. In the current analysis, we found that patients carrying either DRB1*15 or HLA-Cw7 suffered worse OS while patients with either HLA-Cw6 or HLA-B44 had better OS. However, only Cw6 and DRB1*15 could inversely predict RFS (p = 0.021, HR 0.593 and p = 0.026, 1.543 respectively).

As high throughput and high resolution technologies are applied in human study, increasing evidence suggests that a given immune physiological and pathological condition is regulated and governed by coordinated gene networks and overlapping pathways [44]–[46]. Million years of evolution and selection acquired redundant machineries to compensate for single allele variants such as synonymous polymorphism or silent mutation that alone would impact of critical functions. This may have resulted in not one single gene playing a high weight in determining the fate of complex diseases, but rather a combination of modifiers affecting a giving phenotype. Thus, multigenic correlations are more likely to be effective in predicting patient outcomes [47], [48]. In this study, the association of SNPs in the CTLA4, microsatellite in FOXP3 and HLA genotype taken as single factors has marginal bearing on survival risk. However, the analytic strategy based on Cox’s proportional hazards model identified a combination that had predictive value on clinical outcome: HLA-B38, HLA-C15, HLA-Cw3, DRB1*15 and CTLA4 CT60*G/G gene polymorphisms.

Among the five identified markers, HLA-B38, HLA-C15 and HLA-C3 were associated with low risk of RFS. HLA-B38 previously had been positively associated with several autoimmune disorders [49] and negatively with childhood acute leukemia [50].

HLA-Cw3 has been reported as a favorable prognostic biomarker. In a prospective randomized, observation-controlled, phase III trial of adjuvant Melacine as allogeneic stage IV melanoma vaccine study, patients expressing ≥2 specific class I antigens (HLA-A2, HLA-A28, HLAB44, HLA-B45 and HLA-C3) and carrying HLA-Cw3 and/or HLA-A2 genotype enjoyed significant benefit from adjuvant therapy (5-year RFS for vaccinated patients was 77%, compared with 64% in the observation group, *P = 0*.004) [51].

Conversely, the DRB1*15 allele was found to be a high risk marker in our prediction model. DRB1*15 is one of the susceptibility factors for multiple sclerosis [52] and DRB1*15 alleles is associated with significantly increased risk to develop hepatocellular carcinoma in Asians (OR = 2.88, 95%CI: 1.77–4.69, P<0.001) [53]. The DRB1*15-DQB1*06 haplotype is also associated with predisposition to suffer HPV infection (p(c) <0.05) and develop cervical cancer (p(c) <0.05) [54] suggesting that this genotype is associated with malignance predisposition.

In addition to HLA, CT 60 G/G allele, one of the CTLA4 polymorphisms was identified as a high risk markers in this multivariate analysis associated with shorter OS. CTLA4 polymorphism has been reported in association with degree of responsiveness in melanoma patient treated by CTL-4 blockade [55]. CTLA4 haplotypes has also been documented in association with susceptibility to develop esophageal squamous cell carcinoma and osteosarcoma [56], [57]. However, no significant association between CT 60 G/G allele and with risk of developing cancer has been previously reported (CT60: OR, 1.02; 95% CI, 0.80–1.29 for AA + AG vs GG) based on 48 case-control meta analysis studies from 27 articles were analyzed [58].

### Conclusion

Our study suggests that single nucleotide variants and their association with a given condition should be explored together with polymorphisms genes with relevant and compensatory function that are thought to contribute to biological changes. Genome wide association analysis would be an ideal way to explore the complexity of genetic variants associated with disease predisposition, prognosis and treatment response, if patient population and funding issue is not challenged. The five markers identified in the study need to be validated prospectively in an independent cohort of patients receiving the same therapy. Moreover, all patients underwent IFNa treatment following excision of the melanoma but had different survival outcomes; thus, the study was aimed at testing the genetic bearing within the cancer population on clinical outcome. A population not undergoing this treatment is currently not available to these investigators and, therefore, this study cannot conclusively address the issue of whether the combination of genetic markers is predictive or response to therapy rather than reflecting an overall genetic predisposition to better prognosis.

## Supporting Information

Table S1 **Univariate survival analysis for FoxP3 microsatellites.**
(DOCX)
