# Supplementary material for: A Multi-Factorial Genetic Model for Prognostic Assessment of High Risk Melanoma Patients Receiving Adjuvant Interferon
Source: PLoS One. 2012 Jul 24;7(7):e40805. doi: 10.1371/journal.pone.0040805 (PMC3404079; doi:10.1371/journal.pone.0040805)
Supplement: Table S1 — Univariate survival analysis for FoxP3 microsatellites. (DOCX) [file pone.0040805.s001.docx]

**Supplementary table S1. Univariate survival analysis for FoxP3 microsatellites***.

Overall Survival:

| **Variables** | **P value** | **HR** | **95% CI of HR** |
| --- | --- | --- | --- |
| Allele1 E | 0.38 | 0.828 | [ 0.544, 1.258 ] |
| Allele1 O | 0.42 | 1.263 | [ 0.715, 2.232 ] |
| Allele2 E | 0.81 | 1.051 | [ 0.694, 1.592 ] |
| Allele2 O | 0.24 | 0.732 | [ 0.436, 1.228 ] |

RFS:

| **Variables** | **P value** | **HR** | **95% CI of HR** |
| --- | --- | --- | --- |
| Allele1 E | 0.26 | 0.823 | [ 0.586, 1.154 ] |
| Allele1 O | 0.65 | 0.891 | [ 0.536, 1.479 ] |
| Allele2 E | 0.14 | 0.774 | [ 0.553, 1.084 ] |
| Allele2 O | 0.45 | 0.856 | [ 0.574, 1.276 ] |

*Of all potential variant microsatellites, we only include those presented at least with a frequency of 10% of the sample size (i.e., 10% of 284).
